# Evapotranspiration Partitioning of the *Populus euphratica* Forest Ecosystem in the Drylands of Northwestern China

**DOI:** 10.3390/plants14050680

**Published:** 2025-02-22

**Authors:** Qi Zhang, Qi Feng, Yonghong Su, Cuo Jian

**Affiliations:** 1 Key Laboratory of Ecohydrology of Inland River Basin, Northwest Institute of Eco-Environment and Resources, Chinese Academy of Sciences, Lanzhou 730000, China; zhangqi206@mails.ucas.ac.cn (Q.Z.); jiancuo23@mails.ucas.ac.cn (C.J.); 2 University of Chinese Academy of Sciences, Beijing 100049, China

**Keywords:** evapotranspiration (ET) components, water use efficiency (WUE), desert riparian forest ecosystem, seasonal variations, environmental factors

## Abstract

The comprehension of seasonal patterns of evapotranspiration (ET), as well as the interactive response to environmental factors, holds paramount importance for illuminating the intricate interaction within the carbon–water cycle of desert riparian forest ecosystems. Nonetheless, the driving mechanism behind ET changes is complex, and different components show significant differences in response to the same factor. Moreover, water resources are scarce in the region, and sustainable water resources management in arid regions usually aims to maximize transpiration (T) and minimize evaporation (E); therefore, reasonable calculation of ET components is urgent to effectively assess water resources consumption and improve water use efficiency. This discussion assessed the suitability and reliability of different methods for partitioning ET within the desert oasis in Northwestern China, calculated water use efficiency (WUE), and explored the differences in the response patterns of ET, transpiration (T), and WUE to environmental elements of constructive *Populus euphratica* forests in this region during the growing season. Continuous measurements of meteorological, soil, and vegetation factors were collected from 2014 to 2021 to facilitate this investigation. This study demonstrated that the underlying water use efficiency (uWUE) method effectively partitions ET into vegetation T and soil evaporation (E). Seasonal variations in ET and T were predominantly driven by temperature (Ta), radiation (Rn), soil moisture, and leaf area index (LAI). In addition, the exchange of water and carbon across different scales was governed by distinct regulatory mechanisms, where canopy-level WUE (WUEc) primarily depended on climatic conditions, while ecosystem-level WUE (WUEe) was more strongly influenced by vegetation structural characteristics. This study provided valuable insights into the ET characteristics, influencing factors, and water–carbon consumption mechanisms of desert vegetation in arid regions, and the conclusions of the discussion may provide theoretical insights for policymakers and ecosystem managers interested in preserving the ecological balance of arid regions.

## 1. Introduction

Drylands are found in most of the world’s biomes and climatic zones, accounting for more than 40% of Earth’s land area [1]. These areas are exceedingly fragile and highly susceptible to changes and degradation due to limited water resources, extreme weather events, low soil fertility, and slow recovery from disturbances [2]. Desert riparian forests hold momentous ecological value in the various arid ecosystems, characterized by unique biological community structures and ecological functions that play a crucial role in maintaining ecological balance and biodiversity, regulating climate, and mitigating desertification [3]. However, the fragile natural environment of desert riparian forests means that changes in vegetation cover directly impact ecological stability, making them a key focus for ecologists [4]. The ecohydrological process is the core link of a desert riparian forest ecosystem, which is more sensitive to climate change and human disturbance due to the dependence of the ecosystem on scarce water resources. Therefore, the study of the ecohydrological process can reveal the vulnerability of an ecosystem to environmental change and understand its demand for water resources and utilization patterns [5,6]. Evapotranspiration (ET), which refers to the sum of water vapor flux transported by surface and vegetation to the atmosphere, is the vital variable describing the ecohydrological process in this region, and also the main water loss pathway for desert riparian forests [7,8]. In arid areas, more than 95% of precipitation will return to the atmosphere through ET [9]. Due to the differing processes of ET components, their responses to environmental driving factors vary, making the ET partitioning process quite complex [7,8]. With global warming and the increasing frequency and intensity of regional extreme events, vegetation structure and related environmental factors affecting the ET process have become more complex [6,10,11]. ET variation remains the most uncertain aspect of the water cycle, which in turn impacts water use efficiency (WUE) [10,12,13,14]. For that reason, accurately estimating ET and its components is crucial for describing the water exchange characteristics of vegetated and non-vegetated surfaces (such as bare soil), revealing the mechanisms of ecosystem carbon–water coupling interactions, and guiding vegetation restoration and conservation.

Currently, three mainstream means for partitioning ET exist: in situ measurements, empirical statistical methods, and mathematical models [11,15,16,17]. In situ measurements are subject to limited temporal and spatial scales, and in most cases are used to verify the accuracy of empirical statistical methods and mathematical models [17,18,19,20,21]. Among empirical statistical methods, Zhou et al. [22] pioneered an underlying WUE (uWUE) method. Through examining the interplay between gross primary productivity (GPP), vapor pressure deficit (VPD), and ET, the method captures the dynamic response of vegetation to environmental moisture stress, allowing for the estimation of T within the context of total ET. Mathematical models have been proven to be alternative means for partitioning E and T in different geographic areas. The two-source Shuttleworth and Wallace (SW) model [23] provides a robust framework for dissecting the contributions of E and T to ET, which encapsulates the respective resistances to water diffusion from the canopy and soil surfaces to the atmosphere and is particularly effective in environments characterized by sparse vegetation canopies [24,25,26]. Li et al. [27] introduced an improved version of the SW model, referred to as the Simplified SW (SSW) model, which utilized the Priestley–Taylor (PT) model [28] to calculate E to optimize the structure and parameterization scheme. In addition, several notable improvements and algorithmic refinements were introduced to the Penman–Monteith (PM) model, which involved refining the calculation of aerodynamic and surface resistances, optimizing parameterization schemes, and incorporating additional meteorological or surface input variables better to capture the complexity of energy and water fluxes [10,29,30]. Affected by extreme weather and a harsh environment in arid areas, ET has a strong spatial dependence, and long-term measurement of ET flux in arid regions is challenging to achieve [31]. Most studies primarily utilize empirical–statistical methods or mathematical models combined with eddy covariance data to estimate ET and its components, with actual measured data serving as auxiliary verification [3,4,32,33]. Different methods exhibit limitations in performance evaluation, such as the difficulty in obtaining some model parameters, and the error of model parameters will lead to low accuracy of simulation results and poor universality of models [30]. There is no consensus on which method is more suitable for estimating and partitioning ET in desert riparian forests. To select the most suitable method for estimating and partitioning ET in desert riparian forests, conducting a systematic cross-comparison and analyzing several commonly used methods is meaningful. One of the key focuses and challenges in ET partitioning is understanding the factors affecting the partitioning process [34]. Climate, soil, and vegetation each have distinct impacts on ET partitioning [35,36,37], requiring a deeper understanding of how various environmental factors influence these mechanisms.

ET is closely related to regional water use efficiency, accurate calculation and partitioning of ET is the prerequisite for obtaining WUE at different scales according to the definition [38,39,40], and improving water use efficiency—specifically by increasing transpiration and reducing evaporation—is a commonly desired goal for sustainable water resources management in dry areas [3,41,42]. Therefore, in order to better solve the contradiction between supply and demand of regional water resources, it is reasonable to conduct further research on water use efficiency on the basis of ET. The mechanisms by which driving factors regulate WUE across multiple scales under spatiotemporal heterogeneity remain substantially uncertain [42]. For instance, short-term heat stress may reduce WUE by suppressing stomatal aperture, thereby decreasing both transpiration and photosynthesis, whereas prolonged drought could select for deep-rooted vegetation that enhances WUE through optimized water acquisition pathways [3,43]. At site scales, site-level WUE is primarily governed by root distribution and vertical soil moisture gradients, whereas regional-scale patterns are dominated by river discharge and topographic controls [42]. The complexity is further compounded by the fact that identical environmental factors may exhibit opposite responses in regulating ET and WUE. This divergence fundamentally stems from differential sensitivities to the evaporation-to-transpiration ratio, where ET reflects total water loss, while WUE depends on the proportional balance between productive transpiration and non-productive evaporation. Current research challenges center on quantifying the dynamic interplay between evaporation and transpiration under multi-factor interactions, particularly how vegetation adaptation strategies exert cross-scale feedbacks on carbon–water cycling. Addressing these knowledge gaps is critical for advancing ecological restoration and water resource management in arid regions [3,33,43].

In China, desert riparian forests are primarily found in the lower reaches of the inland rivers in the northwest arid region, where they form natural barriers against wind and sand, playing a crucial role in stabilizing river courses, preventing desert encroachment, ensuring the smooth flow of the main water channels [44]. Since the 1950s, due to factors such as rapid population growth, industrial and agricultural production, and climate change, the contradiction between water supply and demand has become increasingly acute, resulting in a severe ecological crisis in the basin [2,5,6], especially in the downstream reaches where the desert riparian forests have been threatened due to water shortage, so that improving WUE is an urgent local objective [45]. The *Populus euphratica*, a primary deciduous tree species growing in desert riparian forests, plays an indispensable ecological role in combating desertification in the lower reaches of inland rivers due to its excellent drought resistance. Owing to their sensitivity to climatic changes and the recent phenomenon of “warming and humid” in the northwest arid region, further research is needed to understand the impact of hydrological variability on ET components and WUE [44]. We proposed the following hypotheses: 

1. The applicability of different ET partitioning methods varied significantly across arid regions, governed by the synergistic regulation of vegetation dynamics and water stress. 

2. The diurnal variations in ET components and WUE were co-driven by phenological stages and environmental stressors, with their sensitivity to influencing factors directly determining ecosystem resilience. 

Therefore, based on an 8-year (from 2014 to 2021) continuous observation of EC measurements, leaf area index (LAI), meteorological data, and sap flow (only including 2014 and 2015), we compared ET partitioning methods, quantified the ET components and WUE, and examined the driving factors behind diurnal variations during the plant phenology period (from May to September), this study pursued the following specific objectives: (1) to discuss the applicability of the uWUE method, SW model, SSW model, and two-source PM model in partitioning ET in comparison with the measured data; (2) to identify the seasonal patterns of ET components and WUE during the whole growing period; and (3) to disentangle the divergent roles of climatic elements, soil moisture and LAI on ET components and WUE.

## 2. Results

### 2.1. Environmental and Biological Factors

**Figure 1:**
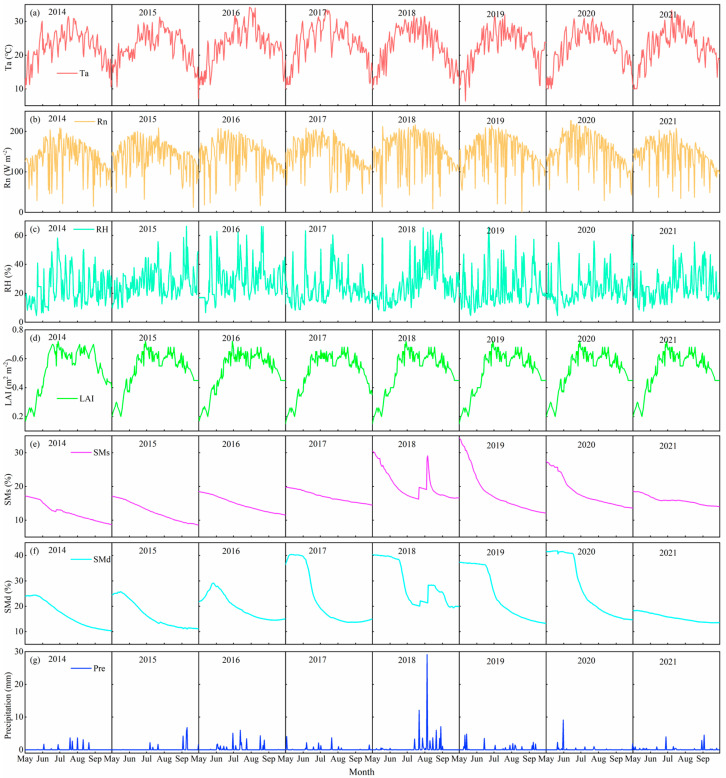
Daily (**a**) air temperature (Ta), (**b**) radiation (Rn), (**c**) relative humidity (RH), (**d**) leaf area index (LAI), (**e**) shallow soil moisture (40 cm, SMs), (**f**) deep soil moisture (200 cm, SMd), and (**g**) precipitation (Pre) from May to September during 2014−2021.

The dynamics of daily air temperature (Ta), net radiation (Rn), relative humidity (RH), LAI, shallow soil moisture content (40 cm, SMs), deep soil moisture content (200 cm, SMd), and precipitation (Pre) during the growing seasons from 2014 to 2021 are depicted in Figure 1. There was little inter-annual difference in Ta, RH, Rn, and LAI, over the multiple years of growing seasons. The daily average Ta and Rn exhibited a consistent unimodal trend and varied from 10.9 to 29.3 °C and 88.6 to 185.8 W m^−2^, with an average of 22.9 °C and 144.1 W m^−2^, respectively, both peaking in July. The multi-year daily mean RH reflected the air dryness, ranging from 12.7 to 40.4%, with an average value of 25.7%. LAI presented an obvious unimodal distribution with Ta and Rn, ranging from 0.2 to 0.7, with a mean value of 0.5, which largely influenced the ratios of E and T to ET through its effects on the transpiration surface area, land cover, and canopy aerodynamics. Except for 2018, both SMs and SMd showed a decreasing trend during the growing season, with great inter-year differences. Shallow soil water was more prone to E, leading to drier conditions in the upper soil layers, whereas deep soil layers were replenished by groundwater, resulting in an SMd that was higher compared to SMs. With the exception of 2018, the growing season precipitation in other years remained within 20–40 mm. Notably, extreme precipitation events occurred in July and August 2018, resulting in a total growing season precipitation of 81.1 mm. The heavy rainfall events (characterized by daily precipitation exceeding 30 mm) during these two months were identified as the primary driver contributing to significant increases in both shallow and deep soil moisture content.

### 2.2. Comparison of the Methods for Partitioning ET

**Figure 2:**
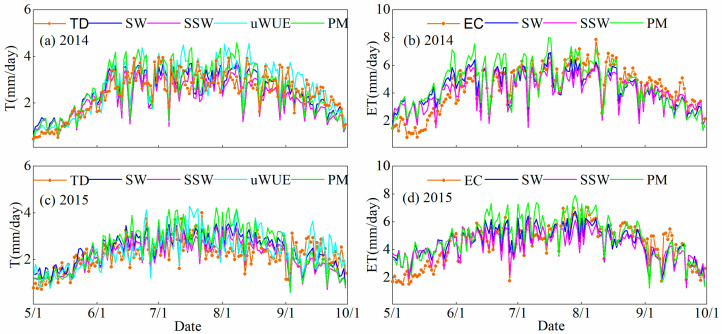
Comparison diagrams between measurement and simulation of transpiration (T) and evapotranspiration (ET) (TD: sap flow; SW: the Shuttleworth and Wallace model; SSW: the Simplified SW model; PM: the two–source Penman–Monteith; uWUE: the underlying water use efficiency method; EC: eddy covariance): (**a**) 2014 T; (**b**) 2014 ET; (**c**) 2015 T, and (**d**) 2015 ET.

**Figure 3:**
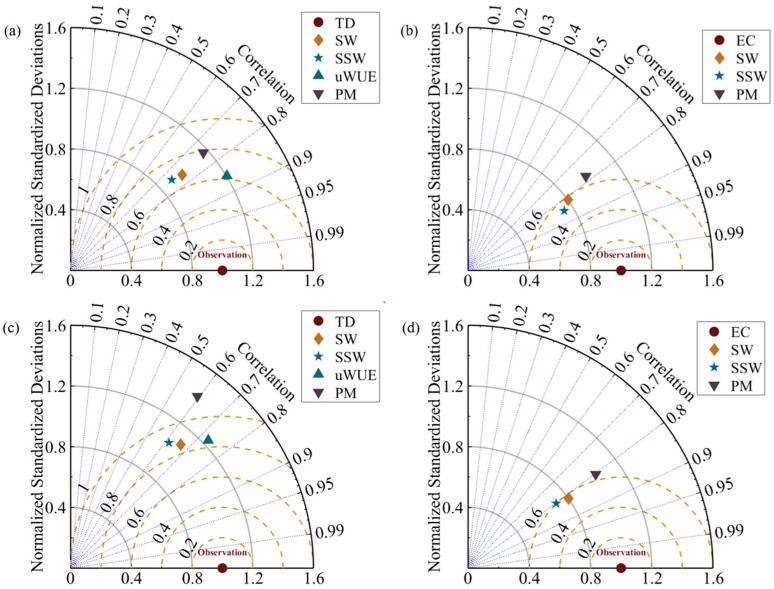
Taylor diagrams between the measurement and simulation of T and ET (TD: sap flow; SW: the SW model; SSW: the SSW model; PM: the two-source PM model; uWUE: the uWUE method; EC: eddy covariance): (**a**) 2014 T; (**b**) 2014 ET; (**c**) 2015 T, and (**d**) 2015 ET.

The calculation of T was conducted using the uWUE method, SW model, SSW model, and two–source PM model, and compared with sap flow values of the 2014 and 2015 growing seasons (Figure 2), then the accuracy of each model was assessed using the Taylor diagrams (Figure 3). As shown in Figure 2a, during the growth period of 2014, the T estimated by the four methods maintained a consistent change trend with the measured data. The uWUE method and the two–source PM model tended to slightly overestimate ET compared to the sap flow method, while the results of the SW model and SSW model were slightly lower than the measured values. Among the four models, the two–source PM model had the highest RMSE at 0.79, followed by the SW model at 0.683, the SSW model at 0.684, and the uWUE model at 0.62 (Figure 3a). Compared to the SW, SSW, and two-source PM models, the uWUE model exhibited reductions in RMSE by 8.6%, 8.8%, and 20.6%, respectively. Moreover, the uWUE method also had the highest correlation coefficient at 0.86, followed closely by the SW model at 0.76, the SSW model at 0.74, and the two-source PM model at 0.75, suggesting very similar estimates of T among these models (Figure 3a). The simulation results for the 2015 growing season also demonstrated a good match with the observed values, with all four methods slightly overestimating T (Figure 2c). The RMSE of the uWUE (0.85) method presented a good consistency with the RMSE of the SW (0.86) model, while the RMSE of the SSW and two-source PM models were relatively higher, at 0.90 and 1.14, respectively (Figure 3c). In terms of the correlation coefficient, the uWUE method was the highest at 0.73, followed by the SW model at 0.66, the SSW model at 0.61, and the two-source PM model at 0.59 (Figure 3c). In summary, the uWUE model performed well in partitioning ET for desert riparian forests and exhibited better agreement with the observed values, while the two-source PM model had larger discrepancies with the measured values.

The SW model, the SSW model, and the two-source PM model can all simulate surface ET, but at the early phase of vegetation growth, all three models significantly overvalued ET compared to the EC method, with the two-source PM model slightly overestimating ET throughout the entire growing season (Figure 2b,d). One account for this deviation in the early growth season was that plants were in the leaf-expansion period with low LAI, causing surface E to be overvalued. Among the three models (Figure 3b,d), the SW model and the SSW model performed better than the two-source PM model (RMSE (2014): SSW (0.54) < SW (0.58) < PM (0.66); RMSE (2015): SW (0.57) < SSW (0.60) < PM (0.64); correlation coefficient (2014): SSW (0.85) > SW (0.81) > PM (0.78); correlation coefficient (2015): SW (0.82) > SSW (0.81) > PM (0.80)). The superior performance of the SW and SSW models compared to the PM model may be related to the determination of aerodynamic impedance. The former divides aerodynamic impedance into two parts—from the soil surface to the canopy height and from the canopy height to the reference height—while the latter considers it as a whole. The SSW model demonstrated higher simulation accuracy, with the advantage of improvements in both model structure and the number of parameters compared to the SW model.

### 2.3. Seasonal Variations in ET, T, WUEc, and WUEe

**Figure 4:**
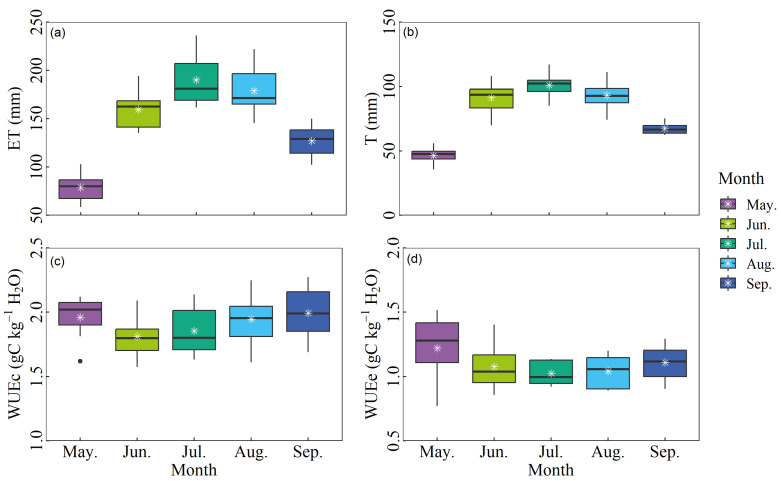
Multi-year monthly average variations of (**a**) ET, (**b**) T, (**c**) canopy WUE (WUEc), and (**d**) ecosystem WUE (WUEe) from May to September.

The comparative results of the models indicated that the uWUE method outperformed other methods in terms of ET partitioning; hence, the calculations of WUEc were based on the results obtained from the uWUE method. The monthly average variations of ET, T, WUEc, and WUEe during the growing season from 2014 to 2021 are depicted in Figure 4, displaying pronounced seasonal differences. During the whole growth period, ET was mainly concentrated from June to August (June: 159.58 ± 19.26 mm, July: 190.05 ± 25.65 mm, August: 178.91 ± 23.46 mm), followed by September (126.93 ± 15.92 mm) and May (79.02 ± 14.11 mm) (Figure 4a), which remained synchronous with the change of T due to the influence of the plant phenology (May: 46.38 ± 6.19 mm, June: 91.16 ± 11.52 mm, July: 101.00 ± 8.70 mm, August: 93.18 ± 10.70 mm, September: 67.53 ± 4.57 mm) (Figure 4b). In ecosystems, WUE reflects both the effectiveness of vegetation in utilizing water resources and its performance in the carbon cycle. This study examined the WUE of the canopy and ecosystem to explore the adaptation strategies of plants to the environment. The range of variation at the monthly timescale in WUEc and WUEe were 1.81–2.04 gC kg^−1^ H_2_O and 1.02–1.22 gC kg^−1^ H_2_O, respectively, which exhibited a similar variation pattern, with the minimum values appearing from June to August, and the maximum values occurring between May and September.

### 2.4. Responses of ET, T, WUEc, and WUEe to Influencing Factors

**Figure 5:**
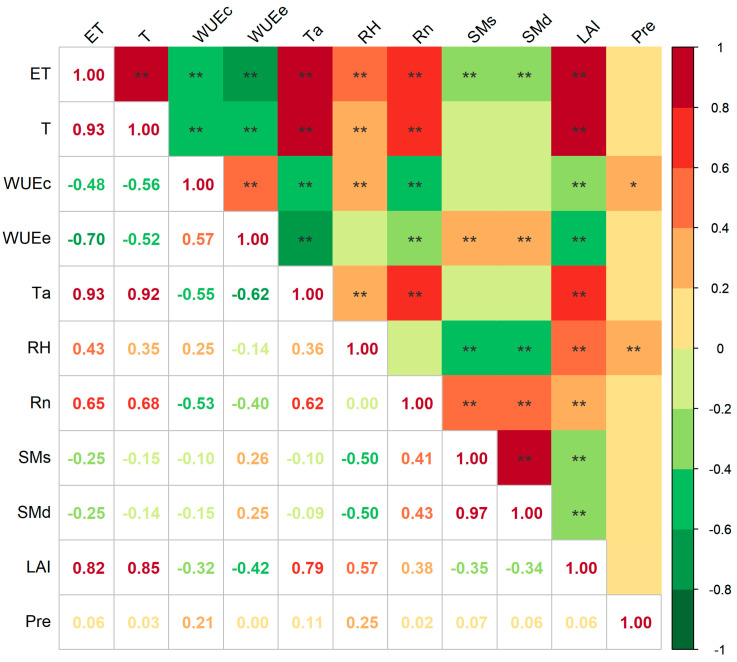
Correlation coefficients of ET, T, WUEc, and WUEe with control factors (** indicates *p* < 0.01, * indicates *p* < 0.05).

**Figure 6:**
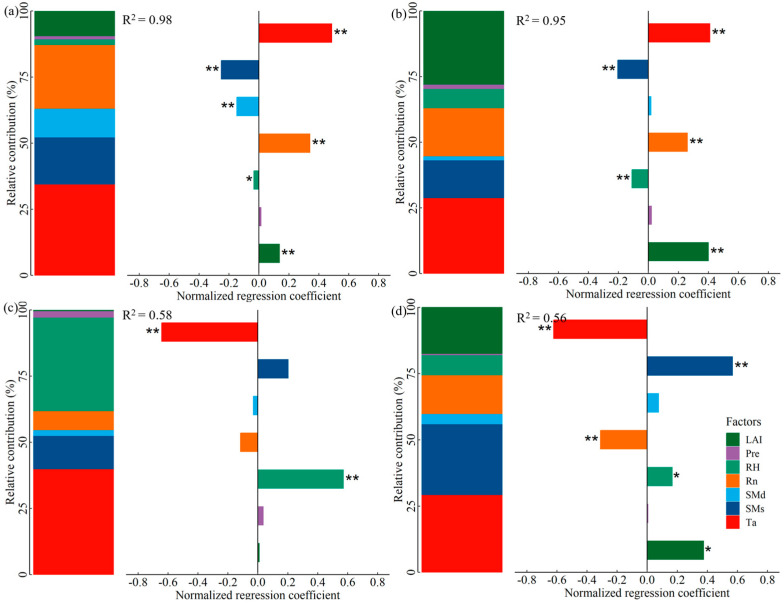
Standardized regression coefficients and relative contribution of environmental parameters to daily variations in (**a**) ET, (**b**) T, (**c**) WUEc, and (**d**) WUEe (* and ** represent significant correlations at 0.05 and 0.01 levels, respectively).

We investigated the influence of meteorological variables (Ta, RH, Rn and Pre), soil conditions (SMs and SMd), and vegetation factor (LAI) at a daily timescale on ET, T, WUEc, and WUEe (Figure 5 and Figure 6). Figure 5 only describes the correlation between ET, T, WUEc, WUEe, and influencing parameters, without quantifying the contribution of different parameters. Meteorological variables and vegetation factors were significantly correlated with ET, T, WUEc, and WUEe. Specifically, ET and T were strongly positively correlated with Ta, RH, Rn, and LAI, while WUEc and WUEe had negative relationships with Ta, Rn, and LAI. Furthermore, WUEc showed a slight positive correlation with RH and Pre, whereas WUEe demonstrated a relatively weak negative relationship with RH (Figure 5). Additionally, the correlation coefficients between ET and T with SMs and SMd were significantly negative. Conversely, the correlation coefficients of WUEc with them are significantly positive, and the relationship of SMs and SMd on the diurnal variation of WUEc was not significant.

We employed multiple linear regression to determine the standardized regression coefficients (absolute values represented the impact of the independent variable on the dependent variable) and relative contribution (RC) of daily variations in Ta, RH, Rn, SMs, SMd, LAI, and Pre (explanatory variables have passed the multicollinearity test) to ET, T, WUEc, and WUEe (Figure 6). ET pronounced a comparatively significant response to Ta, followed by Rn, SMs, SMd and LAI, with RC of 34.51%, 24.12%, 17.86%, 10.48%, and 9.66%, respectively, collectively explaining a 98.01% of variation in ET (Figure 6a). Ta (RC: 28.89%), LAI (RC: 28.15%), and Rn (RC: 18.31%) exerted significant effects on T, while SMs (RC: 14.40%) and RH (RC: 7.67%) were relatively lower, collectively accounting 95.38% of the variability in T by these environmental factors (Figure 6b). WUEc and WUEe demonstrated discrepancies in the responsiveness to environmental conditions, for WUEc, where only Ta (RC: 40.01%) and RH (RC: 35.58%) were the important contributing factors, while WUEe was dominated by Ta (RC: 29.30%), SMs (RC: 26.73%), LAI (RC: 17.71%), and Rn (RC: 14.71% 14.65%), with the control parameters elucidating 57.66% and 55.80% of the changes in WUEc and WUEe, separately (Figure 6c,d).

**Figure 7:**
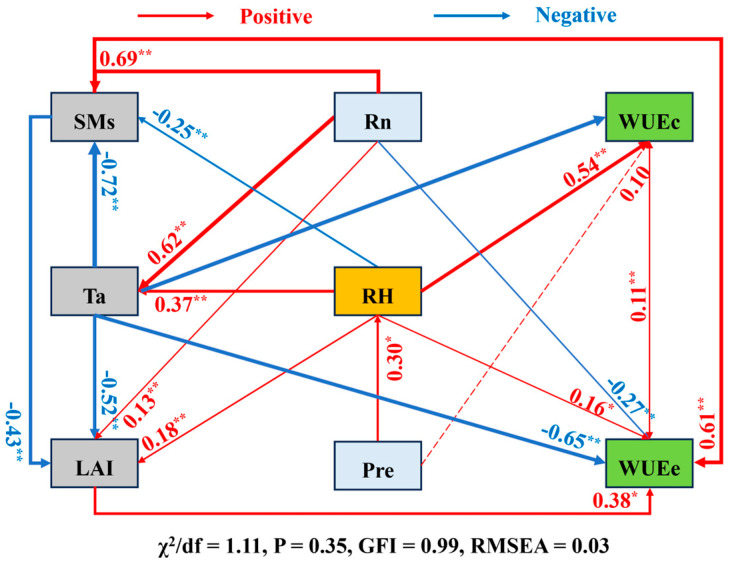
Structural equation model of WUEc, WUEe, and influencing factors (standardized path coefficients are depicted by numbers on the lines, red and blue arrows represent positive and negative correlations, and solid and dashed arrows indicate significant and non−significant relationships, and * and ** represent significant correlations at 0.05 and 0.01 levels, respectively).

Using structural equation modeling (SEM), the direct effects of control parameters on WUEc and WUEe were investigated, along with their indirect effects mediated by other factors, demonstrating that the evaluation indexes of the model were in line with the standards, with a well-constructed model and reliable results (Figure 7). According to the findings, Ta had a considerable adverse effect on both WUEc and WUEe. In contrast, RH had a noteworthy favorable impact on WUEc, though it had a limited effect on WUEe. The direct effects of Pre on WUEc and WUEe were not significant, however, which had indirect effects on WUEc by affecting the RH. Although SMs had a weak influence on WUEc, they exerted a strong direct positive impact on WUEe. Rn indirectly affected WUEc through its impact on Ta, but it had a direct negative effect on WUEc. Finally, LAI had a significant direct positive influence on WUEe.

## 3. Discussion

### 3.1. Comparisons of the ET Partitioning Method

Our study applied the uWUE method, SW model, SSW model, and two−source PM model to partitioning ET, and evaluated the models in combination with ground validation data (Figure 2 and Figure 3). Despite there were some discrepancies between model calculations and sap flow measurements, these methods have been generally implemented in numerous previous studies [3,32,33,46,47]. Each of the four methods has its advantages and disadvantages, and our comparison indicated that the uWUE method provided reliable estimations of T that closely align with observed values. The uWUE method offers simplicity and effectively elucidates the interannual, seasonal, and intraday variation characteristics of T/ET [47]. Furthermore, the estimated source areas of E and T are consistent, eliminating the scale-matching problem [47]. Apart from the desert riparian forest ecosystem, the uWUE method can also effectively calculate the transpiration ratio of desert, meadow, cropland, and shrub ecosystems [33,47,48,49,50]. However, there are two limitations of the uWUE method in practical application. First, this method is suitable for the underlying surface with weak heterogeneity [22]. Second, for the ecosystem with low vegetation coverage and high surface soil water content, the method will lead to the overestimation of T/ET [22], which is also the reason why the uWUE method estimates higher than the sap flow values (Figure 2).

The SW model is primarily used for ET estimation under sparse and uneven vegetation cover, providing a more realistic representation of the physical processes involved in the ET process [51,52,53]. The SSW model optimizes the structural framework of the SW model and simplifies the calculation process using the PT model [27]. Gao et al. [41] evaluated the ET process of *Populus euphratica* in arid areas by using the SW and SSW models, with the findings indicating the superiority of the SSW model in large-scale sparse vegetation systems in extremely arid areas, which was consistent with the ET simulation results in this study. The reason is that the SSW model only uses conventional meteorological data to determine E, while accurately quantifying the resistance parameters in the SW model proves to be more challenging (for example, when calculating soil surface resistance, soil water content should be measured within the range of depth from the soil surface; so far, scholars have not proposed a unified conclusion), which leads to differences in simulation results [27,41]. However, the applicability of SSW model still needs to be further verified. In this study, these two models exhibited similar simulation accuracy on computed T, but comparatively lower goodness of fit compared to the uWUE method (Figure 3). Numerous parameters are involved in the calculation of model resistance, and for ecosystems with tall sparse canopies under extreme drought conditions, the significant short-term variations in various environmental parameters result in environmental instability, leading to differences in time response between simulated and measured results, which is one of the reasons for the observed discrepancies [11,54]. In addition, empirical parameters involved in the formula of Lohammar et al. [55] that we used for canopy stomatal resistance (r_sc_) should be calibrated according to actual conditions. When calculating canopy boundary resistance (r_ac_) using the formula proposed by Choudhury and Monteith [56], the specific conditions such as natural and forced convection are not taken into account, which also impacts the simulation results. Gao et al. [57] pointed out that substantial advective effects caused by wind speed were inconsistent with the assumptions of the model, which could also contribute to the errors. The two-source PM model improves the original model by partitioning the canopy and soil into two independent and interacting source sinks, and incorporating additional meteorological parameters and soil moisture parameters. Wang et al. [3] partitioned the *Populus euphratica* forest ET by using the two-source PM model and found that the simulated and measured T had a high R^2^ value. However, we observed that the two-source PM model exhibited more variability among the four models (Figure 3). This may be because the model often relie on simplified empirical formulations (e.g., surface soil moisture parameterization) for soil evaporation estimation, which inadequately represent moisture dynamics in heterogeneous soils [29,58]. Furthermore, significant errors may arise when aerodynamic resistance parameters (including vegetation canopy and soil resistances) remain unoptimized for sparse vegetation scenarios [58,59].

This paper describes in detail the simulation effects of four common models on ET and its components (such as T and E) in desert riparian forest ecosystems, and discusses the causes of errors, so as to provide references for accurate estimation and segmentation of ET under different scenarios and aid in understanding the variability of surface evapotranspiration components and the ecosystem water cycle process. The simulation results based on eddy covariance parameters are most consistent with the measured data in terms of temporal response, and under the condition of accurately estimating GPP, the uWUE method is undoubtedly the optimal solution for ET partitioning. Both the SW and SSW models show high simulation accuracy with little difference between them in this study, but the SSW model requires fewer meteorological and land surface characteristic data, offering a new option for calculating ET and its components in sparse vegetation canopies in the future. Currently, researchers are focusing on enhancing model usability by accurately quantifying parameters and incorporating site-specific conditions, with the most common approach being the addition of a calibration function to the model. Isotope techniques have been widely used in past research, with stable hydrogen and oxygen isotopes providing valuable insights into the mixing and transport processes of different water bodies [60], making them well-suited for monitoring water movement, and future studies should further explore the integration of models with isotopes.

### 3.2. Effects of Environmental Factors on ET, T, WUEc, and WUEe

In general, the transfer of water, energy, and carbon fluxes within an ecosystem is the result of the synergistic interaction of multiple factors, which collectively determine the rate and direction of transfer [61]. The outcomes of this study revealed that Ta and Rn emerged as the most dominant factors influencing the variation of ET and T. These two factors were found to have an explanatory power of approximately 50% for the variance of ET and T (Figure 6a,b), which was consistent with the conclusions drawn by other scholars [3,33,62]. Since Ta is a critical climate parameter that underpins the migration of matter and the transport of energy at the interface of the atmosphere, soil, and vegetation, as Ta increases, the proportion of Rn converted into ET energy also increases. Furthermore, the energy generated by solar radiation is absorbed by the atmosphere and plants, subsequently influencing the temperatures of both. ET and T were also sensitive to the vegetation factor (LAI) (Figure 6a,b), particularly T [17,63], with the influence of LAI on ET relatively weaker due to the low LAI of the *Populus euphratica* ecosystem in arid areas [22,33]. Soil moisture provided water for plants in desert riparian forests, contributing significantly to both ET and T in this study (Figure 6a,b), as confirmed by [33]. Additionally, relative humidity (RH) had a relatively minor impact on ET and T (Figure 6a,b), which was consistent with the analysis conducted by Yuan et al. [64] on the factors affecting ET in the downstream *Populus euphratica* community of the Tarim River. In summary, the factors affecting ET and its components include meteorological conditions, soil water supply, and vegetation phenology, but the relationships between different ET components and factors like climate and vegetation vary, and their responses to environmental changes are not perfectly synchronized, ultimately influencing the interannual variability of ET. On a seasonal scale, meteorological factors significantly influence the distribution of ET, with temperature changes having the most pronounced effect on ET partitioning. On one hand, temperature affects plant transpiration and soil evaporation by influencing soil moisture levels, but on the other, under high-temperature drought stress, plants can self-regulate in response to environmental conditions to prevent excessive water loss, thereby altering the distribution of ET within the ecosystem. Moreover, some scholars have noted that in arid regions, ET is influenced not only by current climate conditions but also by meteorological factors from the preceding stages of the plant life cycle [65], suggesting that this lagged effect warrants further consideration. In addition to the influencing factors mentioned in this paper, future studies can also systematically assess the contribution of soil texture, canopy structure, and root hydraulic characteristics [66] to the long-term change of ET.

Water use efficiency (WUE) is a crucial metric for comprehending the coupled interactions of water carbon migration and the transformation process, which will be affected due to the distinct responses of the water and carbon cycles to environmental changes [49,67,68]. Previous research has supported that this observation was related to the disparate sensitivities of WUEc and WUEe to variations in influencing parameters [43,69]. Standard regression analysis exhibited that WUEc was primarily regulated by Ta and RH, with minimal influence from other factors (Figure 6c). WUEc and Ta exhibited a notable inverse relationship of statistical significance (Figure 6c), which was probably because high temperature led to a reduction plant enzyme activity, thus suppressing the photosynthetic reaction process of leaves, and resulting in an increase in T at a rate much higher than GPP with increasing Ta [49,70,71]. Additionally, we observed a positive trend contribution of RH to WUEc (Figure 6c). Hu et al. [24] and Li et al. [71] demonstrated that the RH resulted in a reduced humidity difference between the internal and external environments of plant tissues, thereby inhibiting gas diffusion and leading to T reduction. Despite these effects, the influence of RH on photosynthesis was insignificant; therefore, GPP did not change significantly, ultimately leading to a WUEc increase. LAI insignificantly affected WUEc (Figure 6c), which went along with the conclusions of Li et al. [71]. One possible explanation is that LAI affects carbon assimilation and T at the same frequency; that is, as LAI increases, the increases in GGP and T may offset each other, thus suggesting that climatic factors are the primary drivers of WUEc variation [49,72].

The research outcomes supported the perspective that meteorological variables, soil conditions, and vegetation factors jointly determined the pattern of WUEe, which had been reported by other researchers [24,63,73,74,75,76]. The contribution of Ta to WUEe was significantly positive, while RH exhibited a significant negative contribution to WUEe (Figure 6d). The explanations for the impact of Ta and RH on WUEc mentioned earlier were also applicable to WUEe. Rn exerted a significant negative control on WUEe (Figure 6d), consistent with findings from other researchers [77,78]. Li et al. [78] pointed out that plant photosynthesis had a light saturation point, beyond which increased radiation does not further enhance carbon gain but accelerates water consumption, leading to a negative response in WUEe. A relatively strong positive effect of the LAI on the WUEe was found in our study (Figure 6d), presumably because WUEe was calculated by GPP/T * T/ET, which was the product of WUEc and T/ET. Although LAI was not an effective controlling factor for WUEc, former research suggested that T/ET was mainly positively regulated by LAI [49,72,77,79]. Furthermore, soil moisture serves as a principal positive component affecting T/ET in dryland areas [33], together with its insignificant contribution to WUEc [3], which can explain the positive response of WUEe to soil moisture in our results (Figure 6d).

In conclusion, desert riparian forest is a water-scarce ecosystem, and hydroclimatic variability is partly responsible for WUE variation, which has a more apparent effect on canopy WUE than ecosystem WUE. Climate moisture promotes an increase in GPP, with canopy and ecosystem WUE showing positive feedback to atmospheric humidity, while extreme high temperature has the opposite influence. This study found that rainfall events have no obvious influence on the change trend of WUE. This may be because there is little precipitation in this region, with annual precipitation of about 40 mm [80], which is difficult to penetrate underground to replenish water deep in vegetation, and shallow soil water on the surface is easy to evaporate, resulting in a low sensitivity of WUE to small rainfall events. None of the involved natural factors contributed more than 60% to the total variation of WUE, so the impact of human activities on WUE cannot be ignored. The Heihe River Upper Reaches implements ecological water dispatching twice annually to replenish the study area and alleviate local river channel desiccation, but the impact of this measure on the WUE of desert riparian forests remains unclear. The impact of ecological water diversion on WUE in desert riparian forests exhibits a distinct threshold effect. Short-term moderate water supplementation can increase channel runoff and groundwater recharge, compensating for insufficient natural precipitation and alleviating water stress in desert riparian vegetation [81,82]. This intervention enhances plant CO₂ assimilation efficiency and improves photosynthetic rates and net primary productivity (NPP), thereby elevating vegetation water use efficiency [81,82,83]. However, excessive water supplementation may induce root hypoxia, reducing water uptake efficiency and inhibiting photosynthetic activity, ultimately diminishing WUE [84]. Additionally, in arid regions characterized by intense evaporation, water diversion may lead to salt accumulation in surface soils, which could exacerbate osmotic stress on plants and reduce WUE over extended periods [83,84]. Consequently, the influence of ecological water transfer on desert riparian forest WUE constitutes a complex ecohydrological phenomenon involving interactions between plant physiology, hydrological processes, and anthropogenic interventions, which merits further systematic investigation.

## 4. Materials and Methods

### 4.1. Study Site

**Figure 8:**
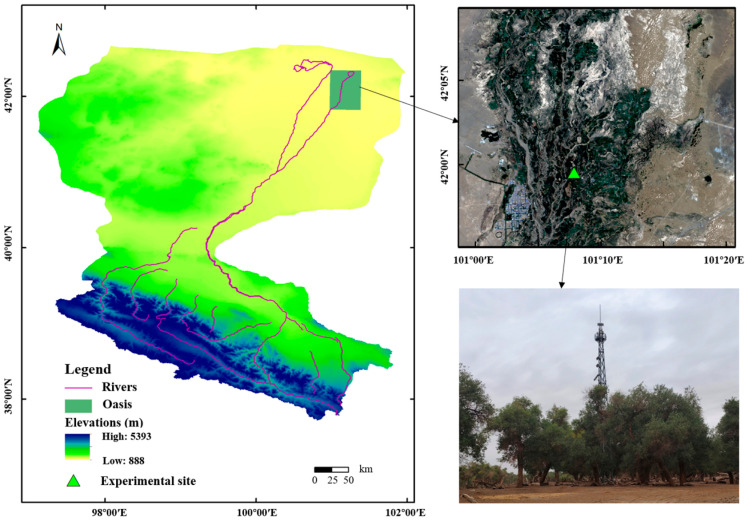
Geographical location of the Heihe River Basin (HRB) and the study area.

The research site (41.99 N, 101.13 E, 876 m) is located in the Sidaoqiao *Populus euphratica* forest, Ejin Oasis, the downstream HRB, Inner Mongolia Autonomous Region, China (Figure 8). The area is alternately controlled by the Mongolian High and Westerlies in winter and summer, respectively, forming a typical arid continental climate, characterized by elevated temperatures and reduced precipitation in summer, followed by cold and dry in winter. Additionally, the area is known for its strong evaporation and frequent sandstorms. According to the meteorological data of Ejin Oasis from 1960 to 2021, the temperature in this area shows an increasing trend, with the annual average temperature of 9.04 °C, the extreme maximum temperature and minimum temperature being 43.1 °C and −37.6 °C, respectively. The annual precipitation is about 41.66 mm, mainly in summer, especially in July and August, and the duration is usually relatively short [80]. However, the potential annual evaporation is about 3755 mm, which is more than 89 times the rainfall [85]. At the site, the mean age of the *Populus euphratica* forest is 80–130 years, with low canopy density and an average height of 13.20 m. The groundwater is deeply buried, and the groundwater level was about 3–4 m during the study period. The soil texture differs with depth, with 0–30 cm being silty loam, 30–100 cm being loam, 100–150 cm being sandy loam, and more than 150 cm being sandy loam.

### 4.2. Measurements and Data Processing

The EC flux tower was installed at the study site to estimate the gas flux of the ecosystem by measuring the gas concentration and vertical wind speed in atmospheric turbulence. In addition to EC observation instruments, meteorological observation instruments, soil heat flux plates, and soil moisture probes were arranged on the flux tower and underground.

The EC observation system requires a flat and uniform underlying surface, with the atmosphere in a turbulent stable state. In practice, these two points are often not satisfied and the original flux data correction is essential. The Eddypro software was used to correct the turbulent data, and the 0.5 h average flux data were obtained after outlier elimination, zero-offset correction, time lag correction, high-pass filtering, co-spectral density correction, coordinate rotation, and seasonal correction [86,87]. Data gaps were interpolated by using the “REddyProc” package in R according to the relationship of incident shortwave radiation (Rg), air temperature (Ta), VPD, and the flux data, along with considering the temporal autoregression of flux data [88,89]. After this processing, the ratio of surface energy flux (LE+H) to available energy (Rn-G) was used as an index to assess the reliability of on-site EC data. A low value of this ratio may indicate potential issues with data quality, necessitating further inspection and correction. The ratio of all years suggested an energy imbalance of approximately 10%, all within a reasonable range (0.55–0.99) [90], and ET was calculated from the Bowen ratio energy balance adjustment of latent heat (LE) [91]. In addition, the GPP was also determined following the standard procedures in “REddyProc” by breaking up Net Ecosystem Exchange (NEE) data [89].

The stem sap flux was measured by the Granier thermal dissipation sap flow velocity probes (TDPs), which were mounted at the trunk diameter breast height (DBH) and oriented in an east–west direction. The original observation data of the TDPs consisted of temperature differences between sensors, which were collected once every 10 s, with an average output of 10 min. Based on the temperature differences, the sap velocity and sap flux were calculated according to the formula proposed by Granier (1987) [92,93,94]. Subsequently, the canopy T was estimated according to the forest area and the distance between trees at the observation point. The leaf area index (LAI) was the product of the Global Land Surface Satellite (GLASS), with a spatial resolution of 250 m and a temporal resolution of 8 days, covering the time range of 2014–2021 [3].

### 4.3. Methods for Partitioning ET

#### 4.3.1. Underlying Water Use Efficiency (uWUE) Method

Zhou et al. [47] proposed a method for partitioning terrestrial ecosystem ET using only 30 min GPP, VPD, and ET data. This method has been widely applied and validated, facilitating research in ET partitioning and ecohydrology. It offers the advantages of simplicity in operation and the ability to decompose ET components within the same source area.

(1) $$\begin{array}{c} u{\mathrm{WUE}}_{p}=\mathrm{GPP}\sqrt{\mathrm{VPD}}/T \end{array}$$

(2) $$\begin{array}{c} u{\mathrm{WUE}}_{a}=\mathrm{GPP}\sqrt{\mathrm{VPD}}/\mathrm{ET} \end{array}$$

(3) $$\begin{array}{c} T/\mathrm{ET}={\mathrm{uWUE}}_{a}/{\mathrm{uWUE}}_{p} \end{array}$$

where uWUEp was determined as the 95th quantile regression between half-hourly GPP·VPD^0.5^ and ET during the whole growing season, and uWUEa at a range of time scales, i.e., daily, was calculated as the linear regression between GPP∙VPD^0.5^ and ET for the corresponding time periods at the site.

#### 4.3.2. The Two-Source Shuttleworth and Wallace (SW) Model

Shuttleworth and Wallace [23] proposed a dual-source ET model of sparse vegetation from the perspective of momentum absorption, energy and material conversion, and material transport, using a resistance network to couple vegetation canopy T and inter-canopy (or sub-canopy) bare soil surface E:

(4) $$\begin{array}{c} ET=E+T=\left(C_{s}PM_{s}+C_{c}PM_{c}\right)/\lambda \end{array}$$

(5) $$\begin{array}{c} {\mathrm{PM}}_{s}=\left\{\Delta A_{c}+\;\left[\rho C_{p}D\;-\Delta r_{a}^{s}\left(A_{c}\;-A_{s}\right)\right]/\left(r_{a}^{a}+r_{a}^{s}\right)\right\}/\left\{\Delta +\gamma \left[1+\;r_{s}^{s}/\left(r_{a}^{s}+r_{a}^{a}\right)\right]\right\} \end{array}$$

(6) $$\begin{array}{c} {\mathrm{PM}}_{c}=\left\{\Delta A_{c}+\left(\rho C_{p}D-\Delta r_{a}^{s}A_{s}\right)/\left(r_{a}^{a}+r_{a}^{c}\right)\right\}/\left\{\Delta +\gamma \left[1+r_{s}^{s}/\left(r_{a}^{c}+r_{a}^{a}\right)\right]\right\} \end{array}$$

(7) $$\begin{array}{c} A_{c}=R_{n}-G \end{array}$$

(8) $$\begin{array}{c} A_{s}=R_{\mathrm{ns}}-G \end{array}$$

(9) $$\begin{array}{c} R_{ns}=R_{n}exp(-0.6LAI) \end{array}$$

(10) $$\begin{array}{c} C_{s}=\left[R_{c}\left(R_{s}+R_{a}\right)\right]/\left(R_{s}R_{c}+R_{c}R_{a}+R_{s}R_{a}\right) \end{array}$$

(11) $$\begin{array}{c} C_{c}=\left[R_{s}\left(R_{c}+R_{a}\right)\right]/\left(R_{s}R_{c}+R_{c}R_{a}+R_{s}R_{a}\right) \end{array}$$

(12) $$\begin{array}{c} R_{a}=\left(\Delta +\gamma \right)r_{a}^{a} \end{array}$$

(13) $$\begin{array}{c} R_{c}=\left(\Delta +\gamma \right)r_{a}^{c}+\gamma r_{s}^{c} \end{array}$$

(14) $$\begin{array}{c} R_{s}=\left(\Delta +\gamma \right)r_{a}^{s}+\gamma r_{s}^{s} \end{array}$$

where C_s_ and C_c_ are the resistance parameters of soil and canopy, respectively; PM_s_ and PM_c_ are the soil and canopy latent heat (W m^−2^) calculated by PM formula, respectively; λ is the latent heat of vaporization of water (2.45 MJ kg^−1^); γ is the psychrometric constant (kPa °C^−1^); Δ is the slope of saturated water vapor pressure with temperature change (kPa K^−1^); ρ is the air density (1.293 kg m^−3^); C_p_ is the specific heat of air at constant pressure (1012 J kg K^−1^); D is the vapor pressure deficit (kPa); A_c_ and A_s_ are the canopy and soil available energy (W m^−2^), respectively; R_n_ is the net radiation (W m^−2^); R_ns_ is the net radiative fluxes into the substrate (W m^−2^); G is the soil heat flux (W m^−2^); $r_{a}^{s}$ is aerodynamic resistance between the soil and canopy height (s m^−1^); $r_{a}^{a}$ is the aerodynamic resistance between the canopy and reference height (s m^−1^); and $r_{s}^{s}$, $r_{a}^{c}$, and $r_{s}^{c}$ are the soil surface, canopy boundary, and stomatal resistance (s m^−1^), respectively.

#### 4.3.3. The Improved Dual-Source (SSW) Model

Li et al. [27] used the PT model (Priestley and Taylor 1972) to calculate E to optimize the parameterization scheme of the SW model:

(15) $$\begin{array}{c} E=\alpha_{E}\tau \left[∆(R_{n}-G)\right]/\left[\lambda \left(\Delta +\gamma \right)\right]\left\{\begin{array}{c} \tau \ll \tau_{c},\alpha_{E}=1\\ \tau >\tau_{c},\alpha_{E}=\alpha -\left(\alpha -1\right)\left(1-\tau \right)\left(1-\tau_{c}\right) \end{array}\right. \end{array}$$

(16) $$\begin{array}{c} T=\left[\Delta R_{\mathrm{nc}}+\left(\rho C_{p}D/r_{a}^{c}\right)+\Delta \left(1-\alpha_{E}\tau \right)\left(R_{n}-G\right)\left(r_{a}^{a}/r_{a}^{c}\right)\right]/\left\{\lambda \left[\Delta +\gamma \left(1+\left(r_{s}^{c}/r_{a}^{c}\right)\right)+\left(\Delta +\gamma \right)\left(r_{a}^{a}/r_{a}^{c}\right)\right]\right\} \end{array}$$

where α_E_ is the PT parameter, τ is the ratio of R_ns_ to R_n_, τ_c_ is the constant (τ_c_ = 0.55), and R_nc_ is the canopy net radiation, and the other parameters have the same meaning as above.

#### 4.3.4. The Two-Source Penman–Monteith (PM) Model

Mu et al. [29] proposed a dual-source structured PM model to estimate E and T in sparse vegetation cover areas. In this model, impedance is divided into soil impedance and vegetation canopy impedance and is combined with the PM formulation through a “series” or “parallel” structure:

(17) $$\begin{array}{c} \lambda ET=\lambda E+\lambda T \end{array}$$

(18) $$\begin{array}{c} \lambda E=\left(\Delta A_{s}+\rho C_{p}D/r_{a}\right)/\left[\Delta +\gamma \left(1+r_{s,s}/r_{a}\right)\right]{\times \left(\mathrm{RH}/100\right)}^{D/\beta } \end{array}$$

(19) $$\begin{array}{c} \lambda T=\left(\Delta A_{c}+\rho C_{p}D/r_{a}\right)/\left[\Delta +\gamma \left(1+r_{s,c}/r_{a}\right)\right] \end{array}$$

where r_s,s_ is the soil surface resistance (s m^−1^), r_a_ is the aerodynamic resistance (s m^−1^), RH is the relative humidity, β is an empirical multiplier [10], r_s,c_ is the canopy stomatal resistance (s m^−1^), and the other parameters have the same meaning as above.

### 4.4. Methods for Calculating WUE

Different definitions and understandings of WUE, as well as different instruments and testing methods used, can lead to different expressions of WUE [21]. The specific formulas for calculating canopy and ecosystem WUE are as follows:

(20) $$\begin{array}{c} {\mathrm{WUE}}_{c}=\mathrm{GPP}/T \end{array}$$

(21) $$\begin{array}{c} {\mathrm{WUE}}_{e}=\mathrm{GPP}/\mathrm{ET}=\left(\mathrm{GPP}⁄T\right)\times \left(T/\mathrm{ET}\right) \end{array}$$

where ET is calculated based on the LE observed by the EC system, GPP is estimated using the “REddyProc” package of R, and T is calculated according to the formula above. The algorithm further reveals the WUEe at the mechanism level, indicating that WUEe is not only controlled by the physiological and ecological processes of vegetation but also by the relative proportion of ET components.

### 4.5. Statistical Analysis

The Taylor diagram is an effective tool for comparing model accuracy, which can cleverly integrate the three model evaluation indices of the correlation coefficient, centered root-mean-square error (RMSE), and standard deviation (SD) into a polar plot according to the cosine relationship between them. In general, scatter points, radiation lines, horizontal and vertical axes, and dashed lines in the Taylor diagram represent the model, correlation coefficient, SD, and centered RMSE, respectively. The correlation coefficient and SD are closer to 1 and the centered RMSE is closer to 0, which means that the simulation result is closer to the observed value and the simulation performance is better. In this study, the standardized Taylor diagram was utilized, where the SD and RMSE of reference and simulated values were divided by the SD of reference values, setting the SD and RMSE of reference values to 1 and 0 and eliminating the physical units.

The Pearson’s correlation coefficient and multiple linear regression model (the data were normalized to eliminate the effects of dimensional and order of magnitude differences) were applied to ascertain how ET, T, WUEc, and WUEe react to related natural parameters throughout the growth period. The standardized regression coefficients in a regression model disclosed the influence level of different variables on the dependent variables [95]:

(22) $$\begin{array}{c} R_{i}=\left|C(i)\right|/\left(|C(\mathrm{Ta})|+|C(\mathrm{Rn})|+|C(\mathrm{RH})|+|C(\mathrm{LAI})|+|C(\mathrm{SMs})|+|C(\mathrm{SMd})|\right) \end{array}$$

where R_i_ is the relative contribution rate of variable i, and C(i) is the standard regression coefficients of variable i.

### 4.6. Structural Equation Model (SEM)

Using the structural equation model (SEM), the complex relationships between variables were evaluated and analyzed, allowing for the simultaneous consideration of both the direct and indirect effects of observed control factors on WUE. The model test criteria are presented in Table 1.

## 5. Conclusions

This study focuses on ET in desert riparian forests, using flux station observation data to discuss the applicability of models for ET partitioning and the driving mechanisms behind the interannual variability of ET components and WUE. In the feasibility verification of the model, we chose four commonly used models to compare the accuracy and explore the causes of errors, among which the uWUE model and SSW model performed best, providing options for ET partitioning under different circumstances. Using temperature, radiation, air humidity, and soil water as abiotic factors, and leaf area index as a biotic factor, partial correlation analysis was used to assess the influence of these factors on the seasonal variations of ET and its components, as well as WUE, and evaluate the contribution rates of the dominant factors. This study found that the driving effects of abiotic and biotic factors on different ET components are not consistent; however, the variations in ET and its components are almost entirely controlled by the synergy of these factors, contributing over 95%. There were differences in the sensitivity of canopy and ecosystem WUE to climate change, with canopy WUE showing a more pronounced response. In terms of vegetation changes, the results indicate that the leaf area index has a weak impact on canopy WUE but is a major controlling factor for ecosystem WUE. The factors mentioned can only explain 60% of the variations in canopy and ecosystem WUE, highlighting that human interventions should not be overlooked. The annual ecological water diversion in the study area alleviates local drought conditions, and its impact on desert riverbank water usage patterns deserves future attention. The ecological environment of desert riparian forests is inherently fragile, with water being the crucial determinant in ensuring ecological stability. Therefore, it is imperative to accurately partition ET and estimate WUE in the area. These computational results contribute to better management of water resources in the region as well as predicting hydrological cycles, which are of utmost importance. The delicate nature of the ecosystem in the area further emphasizes the significance of the precision in these estimates.

## Figures and Tables

**Table 1:** Evaluation metrics of the structural equation model (SEM).

Evaluation Metrics	Chi-Square/df (χ^2^/df)	*p* Value	Comparative Fit Index (CFI)	Root-Mean-Square Error of Approximation (RMSEA)
Standard	<2	>0.05	>0.9	<0.08

## Data Availability

Datasets and other materials are available with the authors, and may be accessible at any time upon request.

## Abbreviations

The following abbreviations are used in this manuscript:

ET	Evapotranspiration
T	Transpiration
E	Evaporation
WUE	Water use efficiency
uWUE	Underlying water use efficiency
WUEc	Canopy water use efficiency
WUEe	Ecosystem water use efficiency
Ta	Temperature
Rn	Radiation
LAI	Leaf area index
RH	Relative humidity
SMs	Shallow soil moisture content
SMd	Deep soil moisture content
Pre	Precipitation
SW	Shuttleworth and Wallace
SSW	Simplified Shuttleworth and Wallace
PM	Penman–Monteith
PT	Priestley–Taylor
EC	Eddy covariance
VPD	Vapor pressure deficit
GPP	Gross primary productivity
